# Supplementary material for: Methylglyoxal Impairs the Pro-Angiogenic Ability of Mouse Adipose-Derived Stem Cells (mADSCs) via a Senescence-Associated Mechanism
Source: Cells. 2023 Jun 28;12(13):1741. doi: 10.3390/cells12131741 (PMC10340470; doi:10.3390/cells12131741)
Supplement: Supplementary file 1 [file cells-12-01741-s001.zip › cells-2374057-supplementary.pdf]

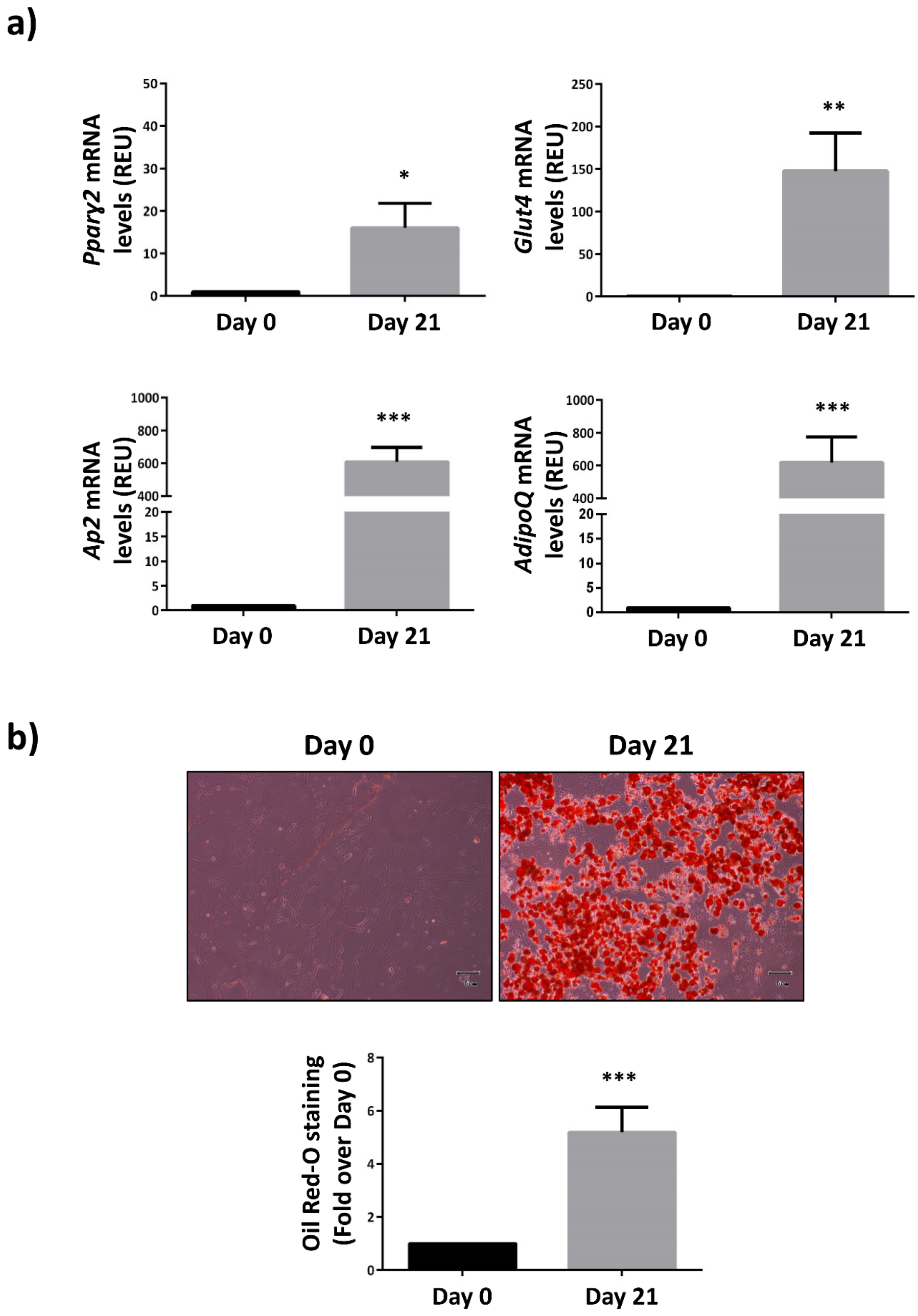

Figure S1: Differentiation of mADSCs in adipocytes. a) mRNA levels of *Pparγ2*, *Glut4*, *Ap2* and *AdipoQ* were measured by qPCR and normalized to *Cyclophilin A* expression in mADSCs before (Day 0) and at the end of differentiation protocol (Day 21); **b)** Representative images of mADSCs (scale bar: 50 μm) stained with Oil Red-O before (Day 0) and at the end of differentiation protocol (Day 21) and the relative quantization. Graphs show the mean ± SEM of 4 independent experiments. Statistical significance was assessed using Student's t-test (* $p \leq 0.05$; ** $p \leq 0.01$; ***$p \leq 0.001$).

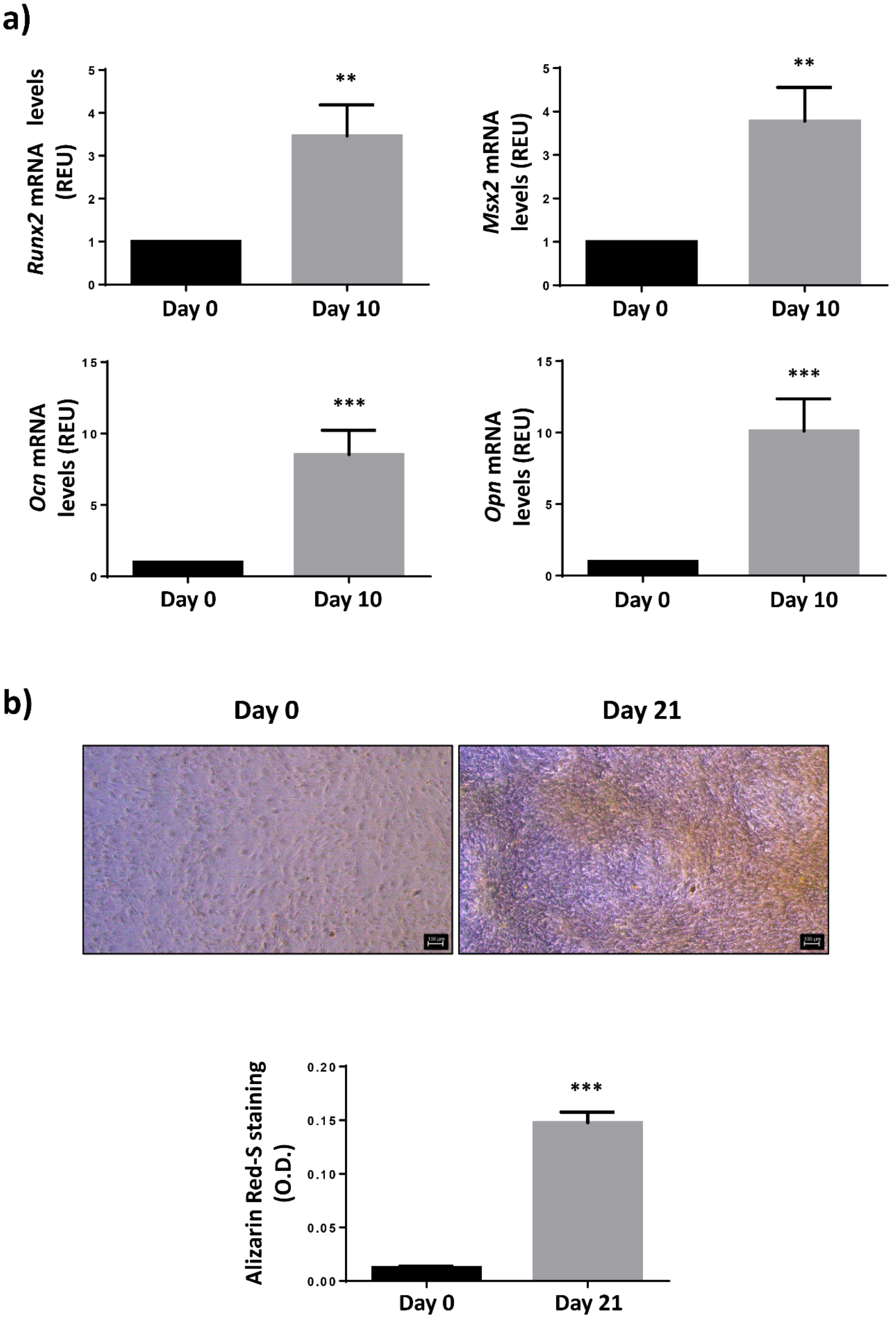

Figure S2: Differentiation of mADSCs in osteoblasts. a) mRNA levels of *Runx2*, *Msx2*, *Ocn* and *Opn* were measured by qPCR and normalized to *Gapdh* expression in mADSCs before (Day 0) and after 10 days of differentiation protocol (Day 10); **b)** Representative images of mADSCs stained with Alizarin Red-S before (Day 0) and at the end of differentiation protocol (Day 21) and the relative quantization. Graphs show the mean ± SEM of at least 3 independent experiments. Statistical significance was assessed using Student's t-test (** $p \leq 0.01$; ***$p \leq 0.001$).
